# AQP2 Promotes Astrocyte Activation by Modulating the TLR4/NFκB-p65 Pathway Following Intracerebral Hemorrhage

**DOI:** 10.3389/fimmu.2022.847360

**Published:** 2022-03-21

**Authors:** Shuwen Deng, Xiqian Chen, Qiang Lei, Wei Lu

**Affiliations:** Department of Neurology, The Second Xiangya Hospital, Central South University, Changsha, China

**Keywords:** aquaporin-2, intracerebral hemorrhage, microglial, astrocyte, immune response

## Abstract

Microglial and astrocyte activation and related cytokine secretion play key roles in secondary brain injury following intracerebral hemorrhage (ICH). We assessed the role of aquaporin (AQP)2 in immune response after ICH. We prospectively collected data from 33 patients with ICH and analyzed the serum AQP2 levels in these patients and age-matched healthy controls. A correlation analysis was also performed between patient serum AQP2 levels and clinical factors. In the rat ICH model, double-fluorescence staining for glial fibrillary acidic protein (GFAP) and AQP2 was performed to investigate the relationship between astrocytes and AQP2. Relative mRNA expression levels of GFAP and AQP2 were also measured. In the rat astrocyte cell line CTX-TNA2, toll-like receptor (TLR)4/nuclear factor kappa B (NFκB)-p65 pathway activation and GFAP levels were measured. The indirect influence of AQP2 on microglial polarization was assessed following exposure to the medium of astrocytes treated with AQP2-overexpression plasmid or silencing RNA. We found that the serum AQP2 expression was lower in patients with ICH. Sex and blood neutrophil count influenced serum AQP2 concentrations in patients with ICH on admission. Lower serum AQP2 levels were inversely correlated with 90-day Modified Rankin Scale scores after ICH, but were not correlated with National Institute of Health stroke scale (NIHSS) scores on admission. AQP2 overexpression and localization in GFAP-labeled astrocytes were observed in rats. AQP2 overexpression induced astrocyte activation with GFAP upregulation *via* TLR/NFκB-p65 signaling pathway activation in the rat astrocyte cell line CTX-TNA2. Astrocyte activation promoted interleukin-1β secretion. The medium of AQP2-overexpression astrocytes promoted the pro-inflammatory M1 phenotype in the immortal rat (HAPI) microglial cell line. Therefore, serum AQP2 is negatively correlated with post-ICH prognosis and may be a marker of inflammation in early-stage ICH. AQP2 overexpression promotes astrocyte activation and pro-inflammatory secretion, affects astrocyte-microglia crosstalk, and indirectly induces microglial polarization, which may augment inflammation after ICH.

## Introduction

Intracerebral hemorrhage (ICH) is a cerebrovascular disease with high mortality and morbidity (1). Hematoma formation leads to mechanical damage to the adjacent tissues during dissection and compression. Under these conditions, multiple blood components (e.g., erythrocytes, leukocytes, and platelets) are released into the brain parenchyma where they can activate microglia, astrocytes, and mast cells through distinct pathways (2). Activated microglia and astrocytes release cytokines, chemokines, prostaglandins, and other immunomodulatory molecules that contribute to secondary brain injury (3). Comprehensive symptomatic treatments, including evacuation of the hematoma by neurosurgical operation and prevention of hematoma expansion by hemostatic therapies (platelet transfusion or recombinant activated factor VII) and intensive antihypertensive treatment, mainly aim to reduce primary injury and have shown only modest efficacy in clinical trials (4). Therefore, there is a need to explore novel approaches to alleviate the adverse effects of neuroinflammation and improve recovery of neurological function after ICH.

Astrocytes are prominent contributors to the immune and inflammatory responses of the central nervous system following ICH. In early stage of ICH, astrocyte activation is evident around the hematoma, presenting with extensive glial fibrillary acidic protein (GFAP) expression (a hallmark of reactive astrocytes) (5). The toll-like receptor 4 (TLR4)/nuclear factor kappa B (NFκB)-p65 pathway-mediated immunomodulatory effects and neuroinflammatory responses contribute to astrocyte activation (6). This activation is accompanied by increased secretion of inflammatory cytokines, such as interleukin (IL)-1β, IL-6, and tumor necrosis factor (TNF)-α, which accelerate the inflammatory response and thus participate in secondary brain injury after ICH (7). Moreover, astrocyte-secreted pro-inflammatory and anti-inflammatory cytokines also influence microglial activation (5, 8). Evidence has demonstrated that crosstalk between astrocytes and microglia, astrocyte-secreted cytokines, transforming growth factor (TGF)-β, IL-33 (9), IL-15 (10), astrocyte-derived chemokines, monocyte chemoattractant protein (MCP)-1/CCL2, and interferon (IFN)-γ inducible protein (IP)-10/CXCL10 can modulate microglial activation and migration (8). Astrocytes can augment the pro-inflammatory response of microglia and release robust levels of inflammatory cytokines, which contribute to severe inflammatory responses and aggravate brain injury (11).

Aquaporins (AQPs) are potential therapeutic targets for alleviating inflammation after ICH, as AQP1, 3, 4, and 8 are involved in the inflammatory process and are considered prognostic markers of lipopolysaccharide (LPS)-induced systemic inflammation (12). In addition to AQPs, AQP2 participates in the regulation of inflammation and apoptosis in renal diseases. As an upstream regulator, AQP2 suppresses LPS-induced renal inflammatory response by reducing the levels of TNF-α, IL-1β, and IL-6 (13). AQP2 also acts as a downstream effector of inflammation; for example, the Nod-like receptor protein 3 (NLRP3)-induced inflammatory response can decrease AQP2 expression in chronic kidney disease (CKD) (14). However, whether AQP2 is expressed in astrocytes and its role in astrocyte activation require further exploration.

This study aimed to understand the potential role of AQP2 in astrocyte activation and immune responses in the brain after ICH and to identify potential therapeutic targets to facilitate clinical therapy research. We also explored the indirect effects of AQP2 overexpression in astrocytes on microglial migration after ICH.

## Materials and Methods

### Clinical Research

#### Patients

This prospective observational study analyzed adult patients with acute, spontaneous, and nontraumatic ICH. We prospectively collected data from patients with ICH admitted to the Second Xiangya Hospital of Central South University. The diagnosis of spontaneous ICH was confirmed based on medical history, computed tomography (CT) scans, and/or brain magnetic resonance imaging (MRI). A total of 43 patients were screened for eligibility between January 1, 2020, and October 31, 2021. Patients were enrolled if they (a) were 18–80 years of age, (b) had a time from symptom onset to admission <24 h, and (c) underwent brain CT scan within 6 h after onset showing spontaneous ICH. Ten patients were excluded for non-spontaneous etiologies of ICH (ICH resulting from aneurysm, antiplatelet or anticoagulant therapy, vascular malformation, tumor, coagulation disorders, or hemorrhagic transformation after cerebral infarction). A total of 33 patients were recruited for this study after screening.

#### Healthy Controls

We also recruited 33 age-matched healthy controls from the Department of Physical Examination at the Second Xiangya Hospital of Central South University. Their health status was assessed using physical examination, laboratory tests, ultrasonography, and imaging.

#### Clinical Data Collection

Data on demographic characteristics (age, sex, blood pressure, and blood glucose), cigarette smoking (at least one cigarette per day for 1 year or more), medication use (anticoagulants, statins, antiplatelet therapy, and antibiotic or immunosuppressant therapy), clinical laboratory tests, imaging (CT and MRI), NIHSS scores on admission, and 90-day Modified Rankin Scale (mRS) scores were collected at the time of enrollment (15). All information was obtained using a standard questionnaire administered by trained staff blinded to the study objectives.

#### Analysis of Serum Levels of AQP2 by Enzyme-Linked Immunosorbent Assay

Blood samples were centrifuged, and serum specimens were collected from patients with ICH within 24 h after ICH onset. The samples were stored at -80°C until their AQP2 concentrations were tested. Commercially available ELISA kits (Cat. # JL34136, Jianglaibio, Shanghai, China) were used to measure AQP2 concentrations within the range of the standard curve. A standard curve was constructed according to the manufacturer’s instructions. The measurement range for the AQP2 kit was 15–480 pmol/L.

#### Ethics Statement

This study was approved by the Ethics Committee of the Second Xiangya Hospital of Central South University. We obtained written informed consent from the patients and healthy controls and handled the patient data and blood samples according to the published International Health Guidelines (Declaration of Helsinki, 2008).

#### Statistical Analysis of Human Data

Categorical variables are presented as percentages (counts), and Fisher’s exact test was performed. Continuous variables were presented as mean (SD) or median (range), as appropriate. Kolmogorov–Smirnov tests were used to determine whether the sample data were normally distributed. Student’s t-tests were performed when the sample data were normally distributed; otherwise, Mann–Whitney U tests were performed. Multiple linear regression analysis was performed to determine the risk factors for serum AQP2 concentration. Serum AQP2 cutoff points were determined using the receiver operating characteristic (ROC) curve and Youden index. Statistical significance was set at p < 0.05.

### ICH Models Established *In Vitro* and *In Vivo*

#### Animals and ICH Model

Adult male Sprague–Dawley rats were used in this study. The rats used for all experiments were six to eight weeks old, weighed 250–300 g, and were age-matched. In the experiment, 36 rats were used (40 rats underwent surgery, and 36 survived) to evaluate the effect of AQP2 expression on early brain injury post-ICH. The rats were randomly and evenly assigned to four groups of nine rats each: sham group, 12 h ICH group, 24 h ICH group, and 48 h ICH group. The rats were sacrificed at 12, 24, and 48 h, and brain tissue samples were collected for qPCR (n = 6) and immunohistochemistry (IHC)/immunofluorescence (IF) (n = 3).

All animal experiments and protocols were approved by the Central South University Animal Experimentation Committee (approval number: 2021013), and were in complete compliance with the National Institutes of Health Guide for the Care and Use of Laboratory Animals (NIH Publications No. 8023, revised 1978).

The rats were kept at room temperature (25°C) and allowed free access to water and food. The light was controlled at 12-h day and night intervals, and the rats were randomly grouped. Rats were anesthetized with isoflurane (cat. #R510-22, RWD, Shenzhen, China) (2%–4%) and placed in a stereotaxic apparatus (cat. #68526, RWD). ICH was induced by infusion of type IV collagenase (cat#C5138, Sigma-Aldrich) through a microinjection pump (cat. #KDS LEGATO 130, RWD) at a rate of 0.4 μL/min, as previously described (16). Collagenase type IV (0.5 U in 2 μL saline) was slowly infused into the right basal ganglia at 0.1 mm anterior-posterior (AP), 3 mm medial-lateral, and 6.0 mm dorsal-ventral to the bregma. Sham-operated rats were administered sterile normal saline. The rats were allowed to recover in separate cages with free access to food and water.

#### Cell Culture and Cell Viability Assay

Primary astrocyte cultures were prepared from the cerebral cortices of 1- to 2-day-old neonatal Sprague–Dawley rats, as described by Grace et al. (17). Primary microglial cells were separated from astrocytes and oligodendrocytes by shaking the flasks on a rotary platform in a 37°C incubator at 200 rpm overnight (17). Primary astrocyte and microglial cells were cultured in Dulbecco’s modified Eagle’s medium (DMEM) containing 10% fetal bovine serum.

The immortalized rat astrocyte cell line CTX-TNA2 was purchased from Shanghai Saibai Kang Biotechnology Company (cat. #iCell-r008, Shanghai, China). The immortalized rat microglial cell line HAPI was purchased from Shanghai Qingqi Biotechnology Company (cat. # BFN60810727, Shanghai, China). CTX-TNA2 and HAPI cells were cultured in DMEM (cat. #11965118; Life Technologies, Carlsbad, CA, USA) supplemented with 10% fetal bovine serum (cat. #16000-044, Gibco, Gaithersburg, MD, USA), and 1% penicillin-streptomycin (cat. #15140122, Gibco) in a humidified 37°C incubator with 5% CO_2_ (18). To establish an *in vitro* ICH model, the CTX-TNA2 and HAPI cell lines were treated with hemin (cat. #51280; Sigma-Aldrich, St. Louis, MO, USA), a degradation product of hemoglobin, which plays a key role in secondary neuronal injury after ICH (19).

The CCK-8 assay was used to determine cell viability (cat. E-CK-A362; Elabscience Biotechnology Co., Ltd. Wuhan, China), at an absorbance of 450 nm, and detected using a microplate reader (Bio-Rad Laboratories, Hercules, CA, USA).

#### Plasmid and siRNA Transfection of Astrocytes

AQP2 was extracted from the astrocyte cell line CTX-TNA2 and cloned into a pcDNA3.1(+) vector. The AQP2-specific primers used were as follows: forward, 5’-CTACCGGACTCAGATCTCGAGGCCACCatgtgggaactccggtccatagcgttctcccg-3’ and reverse 5’-GTACCGTCGACTGCAGAATTCGAggccttgctgccgcgcggcaggctctgcggagagtg-3’. After 24 h of transfection, astrocytes were subsequently exposed to hemin and harvested at 48 h for further analysis. We used PepMute (SignaGen, Frederick, MD, USA) to transfect siRNA-targeting AQP2 (si‐AQP2) or negative control-siRNA (NC-siRNA) into astrocytes for 48 h. Astrocytes that were treated with si-AQP2 or siRNA-NC were exposed to hemin for 24 h and harvested at 48 h for further analyses. Microglia were treated with the medium of astrocytes transfected with or without hemin.

TAK-242, a TLR4 inhibitor (cat. #243984-11-4; MedChemExpress, Shanghai, China) was administered to astrocytes. The astrocytes were treated with TAK-242 (300 nM) after pcDNA3.1-AQP2 plasmid transfection for 24 h and harvested after 48h. Microglia were cultured in the harvested astrocyte medium for 24 h.

#### Immunofluorescence and Western Blot Analyses

IF staining was performed using anti-AQP2 antibody (1:100 dilution; cat. #A16209; ABclonal Technology, Wuhan, China), anti-GFAP (1:1000; cat. #60190-1-Ig; Proteintech, Wuhan East Lake, China), and anti-CD68 (1:100 dilution; cat. #ab201340; Abcam, Cambridge, UK), as previously described (20). The secondary antibodies were conjugated to Alexa Fluor 594 goat anti-mouse IgG (1:400 dilution; cat. #A-11030, Invitrogen) and Alexa Fluor 488 goat anti-rat IgG (1:200 dilution; cat. #A-11034; Invitrogen), corresponding to the primary antibodies. Nuclei were stained with 4′,6-diamidino-2-phenylindole (DAPI) (cat. #28718-90-3, Sigma-Aldrich).

For immunoblot analysis, the protein concentration in the supernatant was determined using a bicinchoninic acid assay kit (cat. # ZJ101; Shanghai Epizyme Biomedical Technology Co., Ltd., China). Forty micrograms of proteins from each sample were separated using sodium dodecyl sulfate-polyacrylamide gel electrophoresis and transferred to polyvinylidene difluoride membranes, which were blocked with Tris-buffered saline containing 5% skim milk for 1h at 25°C. WB was performed by overnight incubation at 4°C with the corresponding primary antibodies, including anti-AQP2 (1:1000 dilution; cat. #A16209; ABclonal Technology, Wuhan, China), anti-GFAP (1:1000; cat. #60190-1-Ig; Proteintech, Wuhan East Lake, China), anti-IL-β (1:1000 dilution; cat. #16806-1-AP; Proteintech, Wuhan East Lake, China), anti-TLR4(1:1000 dilution; cat. #19811-1-AP; Proteintech, Wuhan East Lake, China), anti-NFκB-p65/p65 (1:1000; cat. #66535-1-Ig; Proteintech, Wuhan East Lake, China), anti-phospho-NF-κB p65 (S536) (1:300 dilution; Cat# GB113882; Wuhan Servicebio Technology Co., Ltd., China), anti-mannose receptor/CD206 (1:1000 dilution; cat. #ab64693; Abcam, Cambridge, UK), anti-CD68 (1:100; cat. #ab201340; Abcam, Cambridge, UK), anti-CD86 (1:100; cat. #sc28347; Santa Cruz Biotechnology, Texas, USA), and anti-β-actin (1:1000 dilution; cat. #66009-1-Ig, Proteintech, Wuhan East Lake, China). β-Actin was used as an internal control to normalize the loaded proteins. The blots were incubated with secondary antibodies, including horseradish peroxidase (HRP)-conjugated AffiniPure goat anti-mouse IgG(H+L) (cat. #SA00001-1, Proteintech) or HRP-conjugated AffiniPure goat anti-rabbit IgG(H+L) (cat. #SA00001-1, Proteintech). The protein bands were visualized using enhanced chemiluminescence (ECL) (LI-COR ink, USA), and the gray values of the bands were analyzed using ImageJ software (National Institutes of Health, USA).

#### Quantitative Real-Time Polymerase Chain Reaction

Total ribonucleic acid (RNA) was extracted from the brain tissues of each group using TRIzol reagent (Invitrogen, Grand Island, NY, USA). RNA (1 µg) was reverse-transcribed into complementary DNA (cDNA) using a cDNA synthesis kit (Invitrogen) according to the manufacturer’s protocol. The relative levels of the messenger RNA (mRNA) transcripts of each gene to those of β-actin were determined by qRT-PCR using a SYBR premixed system and specific primers. The primer sequences were as follows: forward, 5’- TTCCTTCGAGCTGCCTTCTA-3’ and reverse, 5’-TTGTGGAGAGCATTGACAGC-3’ for AQP2 (122 bp); forward, 5’- ATCGAGATCGCCACCTACAG -3’ and reverse, 5’- CTTCTTTGGTGCTTTTGCCCC -3’ for GFAP; and forward 5’- ACATCCGTAAAGACCTCTATGCC -3’ and reverse 5’-TACTCCTGCTTGCTGATCCAC -3’ for β-actin (223 bp). The PCR reactions were performed in triplicates at 95°C for 5 min, followed by 40 cycles at 95°C for 20 s, 72°C for 20 s, 72°C for 5 min, and 55°C for 10 s. The relative mRNA transcript levels of each gene were analyzed using the 2Ct method and normalized to those of β-actin. All experiments were performed in triplicates.

#### Statistical Analysis of Experimental Data

Data are expressed as mean ± standard deviation (SD). One-way analysis of variance was used for comparisons between multiple groups of data, whereas two groups of data were compared using Student’s t-tests. Differences were considered statistically significant at p < 0.05. Data analysis and plotting of figures were performed using Prism 7 software (GraphPad Software Inc., San Diego, CA, USA).

## Results

### Serum AQP2 Levels Are Lower in Patients With ICH Than in Healthy Controls

**Figure 1:**
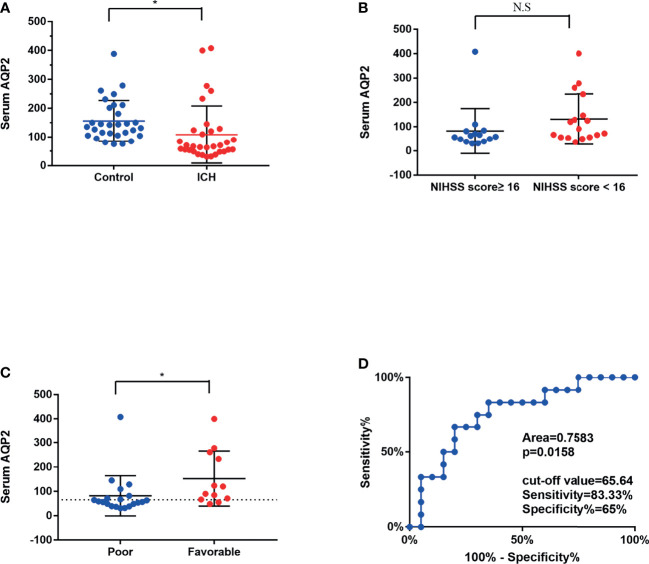
Mean serum AQP2 concentrations in different groups. **(A)** Serum AQP2 concentration had significantly decreased at 1 days after the bleeding in an ICH patient compared to that in a healthy control (*P < 0.05). **(B)** Mean serum AQP2 concentrations was presented no difference between patients with NIHSS scores ≥ 16 and NIHSS scores < 16. **(C)** Mean serum AQP2 concentrations in poor prognosis (mRS scores>3) were significantly lower in comparison to those in favorable prognosis (mRS scores ≤ 3) (*P < 0.05). **(D)** ROC curves of serum AQP2 concentrations for predicting different prognosis.

The ICH group comprised 21 men and 12 women with a mean age (SD) of 60.9 (7.1) years. The control group included 17 men and 16 women, with a mean age (SD) of 61 (10.3) years. The read points of measurement of AQP2 concentration in one ICH patient and three healthy controls were more than 500 pmol/L, and these data were excluded from this study. As shown in **Figure 1A**, serum AQP2 concentration in patients with ICH (n=32, 108.54 ± 17.61 pmol/L) was lower than that in healthy controls (n=30, 156.22 ± 13.01 pmol/L) (p < 0.05). The demographic characteristics of the two groups are shown in **Table 1**. Patients with ICH showed higher morbidities of smoking, alcohol consumption, and hypertension than healthy controls.

**Table 1:** Demographic characteristics of patients with ICH and healthy controls.

Variables	ICH patients (n=33)	Healthy Control (n=33)	p value
Age, year, mean (SD)	60.9 (7.1)	61.0 (10.3)	0.945
Sex, n (%)			0.455
Male	21 (63.6%)	17 (51.5%)	
Female	12 (36.4%)	16 (48.5%)	
Hypertension, n (%)	29 (84%)	18 (54.5%)	0.006^*^
Hypercholesterolemia, n (%)	11 (33.3%)	13 (39.4%)	0.798
Diabetes mellitus, n (%)	8 (24.2%)	12 (36.4%)	0.422
Smoking, n (%)	24 (72.7%)	10 (30.3%)	0.001^*^
Alcohol consumption, n (%)	21 (63.6%)	9 (27.3%)	0.006^*^
Aspirin use before admission, n (%)	8 (24.2%)	10 (30.3%)	0.783
Statin use before admission, n (%)	10 (30.3%)	12 (36.4%)	0.794

*p < 0.05.

### Variables Associated With Serum AQP2 Levels of Patients With ICH on Admission

Multivariate linear regression analyses indicated that serum AQP2 concentration was mainly related to sex and blood neutrophil count, while other variables showed no difference (**Table 2**). The linear regression formula was y = 86.077 x1 + 14.014 x2 + 27.752, where y, x1, and x2 represent serum AQP2 concentration, sex, and blood neutrophil counts, respectively.

**Table 2:** Multivariate linear regression analysis of the risk factors for AQP2 in patients with ICH (n=32).

Variable	Value	p value
Age, Mean (SD)	60.5 (10.0)	0.290
Sex (Male, %)	20 (62.5%)	0.026^*^
Blood platelet counts, Mean (SD)	228.22 (97.08)	0.193
Blood neutrophils counts, Mean (SD)	8.03 (2.91)	0.029^*^
Blood lymphocytes counts, Mean (SD)	1.30 (0.80)	0.824
Erythrocyte sedimentation rate, Mean (SD)	38.06 (29.96)	0.390
Extension to ventricles (%)	11 (34.3%)	0.164
Serum Procalcitonin, Mean (SD)	0.15 (0.20)	0.227

*p < 0.05.

#### Relationships of Serum AQP2 Concentration With Disease Severity and Prognosis

The mean serum AQP2 concentrations did not differ significantly between patients with NIHSS scores ≥16 and those with NIHSS scores <16 (82.02 ± 18.51 pmol/L vs. 152.73 ± 32.57 pmol/L, p >0.05) (**Figure 1B**). The mean serum AQP2 concentrations in patients with a poor prognosis (mRS scores >3) were significantly lower compared to those with a favorable prognosis (mRS scores ≤3) (82.56 ± 23.93 pmol/L vs. 131.46 ± 24.88 pmol/L, p < 0.05) (**Figure 1C**). The ROC curves for serum AQP2 concentrations in ICH patients with different prognoses are shown in Fig. 1D. The area under the curve for poor prognosis was 0.7583. Based on the ROC curve, the optimal serum AQP2 cut-off was 65.64 pmol/L.

#### Significant AQP2 Upregulation in Astrocytes Following ICH in Rats

**Figure 2:**
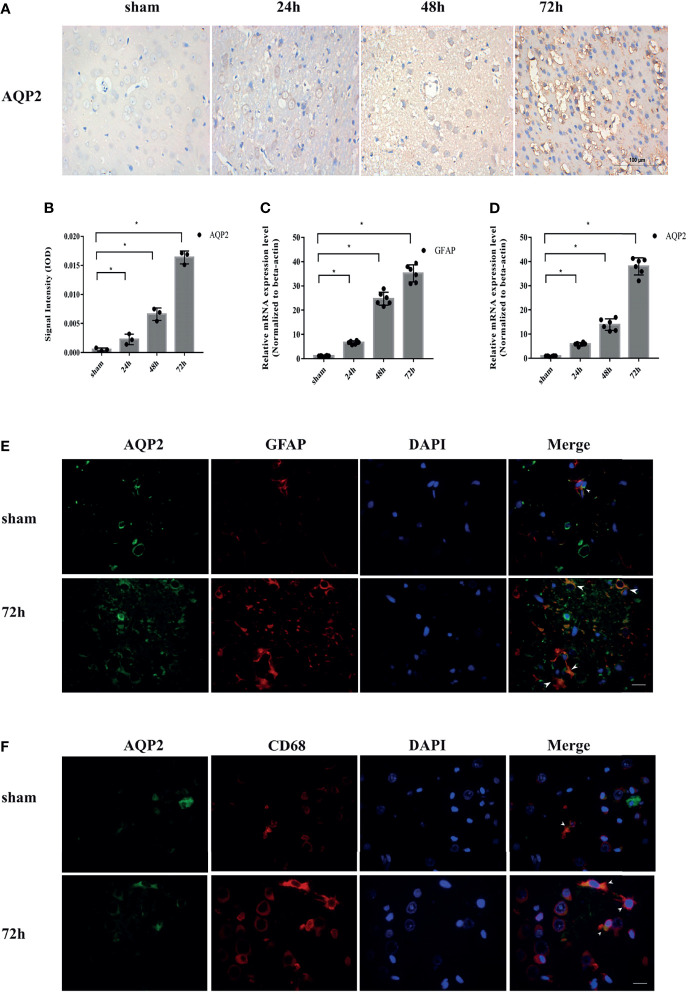
AQP2 is highly upregulated after ICH in rat brain induced by ICH. **(A)** Immunohistochemical staining of AQP2 in the perihematomal area of hemorrhaged brain in rats (Scale bar=100um). **(B)** Data of integrated optical density (IOD) value shown as mean ± SD, were revealed a higher expression of AQP2 around hematoma in ICH groups (after 24h, 48h or 72h) compared with sham group (n=3, *p<0.05). Quantification of the relative mRNA expression of AQP2 **(C)** and GFAP **(D)** in basal ganglia region of rat brain in sham group or in perihematoma area of hemorrhaged brain after ICH (n=6, *p<0.05). Double fluorescence staining AQP2 and astrocyte marker GFAP **(E)** or M1 phenotype marker CD68 **(F)** revealed the co-localization of AQP2 and astrocyte or microglial in basal ganglia region of rat brain in sham group or in perihematoma area of hemorrhaged brain after ICH (white arrows) (Scale bar=50um).

We assessed the expression of AQP2 in astrocytes in a collagenase-induced rat ICH model. Immunohistochemical staining revealed increased AQP2 expression in the perihematomal area of the hemorrhaged rat brain (**Figures 2A, B**) (p <0.05). Fluorescence staining was used to examine the localization of AQP2 in astrocytes or microglia in the sham group (around the basal ganglia region of the rat brain) or in the ICH group (perihematoma area of the hemorrhaged brain). Double fluorescence staining of AQP2 and the astrocyte marker GFAP or microglial M1 phenotype marker CD68 revealed that AQP2 was co-localized with astrocytes and microglia (**Figures 2E, F**), in which both AQP2 and GFAP/CD68 were overexpressed in the hemorrhaged rat brain. In addition, qPCR analysis of the brain tissues obtained from the sham and ICH groups (n=6) further supported these results (**Figures 2C, D**) (p <0.05).

### AQP2 Is Highly Expressed, and the Inflammation Response Is Increased In Astrocytes in Hemin-Induced ICH *In Vitro*

To investigate the localization of AQP2 in specific cells in the central nervous system (CNS), we explored whether AQP2 was expressed in C6 (glioma cells), rat BMECs (brain microvascular endothelial cells), CTX-TNA2 (astrocytes), and HAPI (microglia) cells. AQP2 expression was detected in glioma cells, astrocytes, and microglial cell lines, but was rarely expressed in rat BMECs (**Supplementary Figure 1A**).

We first investigated the effect of hemin on primary astrocytes and microglia using an ICH model *in vitro*. The protein expression levels of AQP2, GFAP, TLR4, NF-κB (p65), and IL-1β were upregulated after hemin treatment at concentrations of 25 µM, 50 µM, and 75 µM compared to those in the normal group (**Supplementary Figure 2A, C–E, G, H**) (p < 0.05). AQP2 was mainly located in the cytoplasm and cell membranes of the primary astrocytes (**Supplementary Figure 2B**). Moreover, the M1 phenotype was increased in primary microglia cultured with the medium of astrocytes treated with hemin (25-75 µmol/L), as indicated by CD68 upregulation (**Supplementary Figure 2F, I**, p < 0.05). Although the M2 phenotype marked with CD206 was also increased in primary microglia treated with 50 and 75 µmol/L hemin (**Supplementary Figure 2J**), the ratio of the M1/M2 phenotype was also increased under these conditions (**Supplementary Figure 2K**) (p < 0.05).

**Figure 3:**
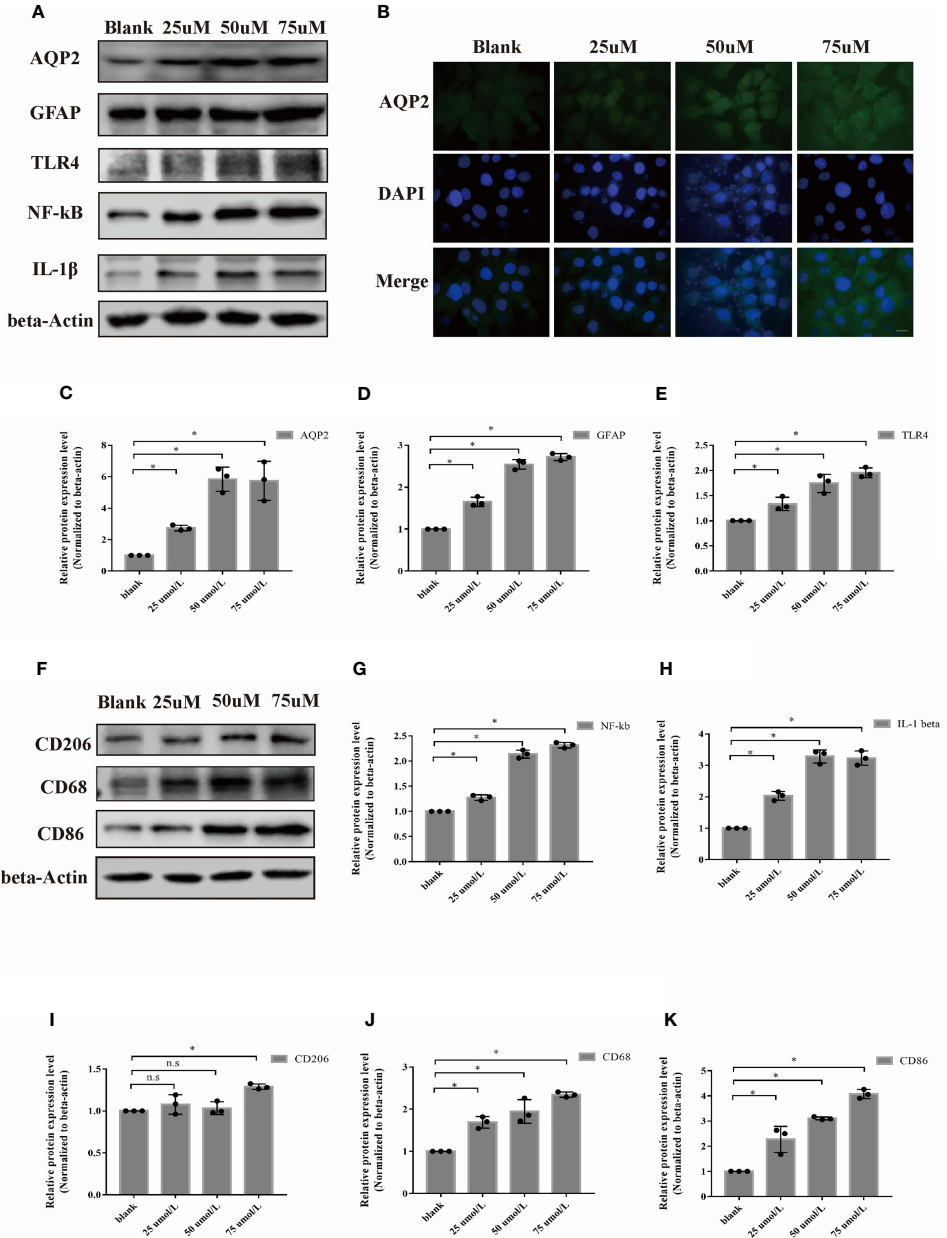
Hemin induced astrocyte activation and microglia polarization. Astrocyte was exposed to hemin for 24 h. **(A)** AQP2, GFAP, TLR4, NF-kB and IL-1β protein levels in rat astrocyte cell line CTX-TNA2 were assessed by western blotting in the blank control group, and different groups were treated with several concentrations of hemin (n = 3/group). **(B)** Astrocyte exposed to hemin for 24 h were stained with antibodies to AQP2 (green signal) and visualized by immunofluorescence microscopy. Astrocyte were immunostained for nuclei (blue, DAPI). Representative images are shown. Scale bar: 10 μm. Quantification of the relative protein expression level of AQP2(C), GFAP **(D)**, TLR4 **(E)**, NF-kB **(G)** and IL-β **(H)** after 24 h hemin treatment. *p < 0.05 vs. the blank group without hemin. **(F)** CD206, CD68 and CD86 protein levels in microglia in the blank control group were assessed by western blotting, and different groups were treated with several concentrations of hemin (n = 3/group). Quantification of the relative protein expression level of CD206 **(I)**, CD68 **(J)** and CD86 **(K)** are shown. *p < 0.05 vs. the blank group without hemin. The data are presented as the mean ± s.d. of three independent experiments.

We selected the rat astrocyte cell line CTX-TNA2 to further study the potential role of AQP2 in astrocytes. Similar to primary astrocytes, the protein levels of AQP2, TLR4, NF-κB (p65), IL-1β, and GFAP were also upregulated in the CTX-TNA2 group compared to those in the normal group (**Figures 3A** **, C–E, G, H**, p < 0.05). **Figure 3B** supports the observation that AQP2 upregulation is induced by hemin. In addition, CD68 and CD86 was increased in HAPI cultured in the medium of astrocytes treated with hemin (25-75 µmol/L) (**Figures 3F, I–K**, p < 0.05). In contrast to primary microglia, CD206 was increased in HAPI treated with 75 µmol/L hemin, but was not significantly different at 25 and 50 umol/L. The ratio of the M1/M2 phenotype was generally increased (**Figures 3** **F, I**, **Supplementary Figures 1B, C**). Control and treated immortalized astrocytes and microglial cells showed similar responses to hemin compared with primary rat astrocytes and microglial cells (derived from primary astrocytes).

### AQP2 Positively Affects Astrocyte Activation and Indirectly Increases Microglia Transition to the M1 Phenotype

**Figure 4:**
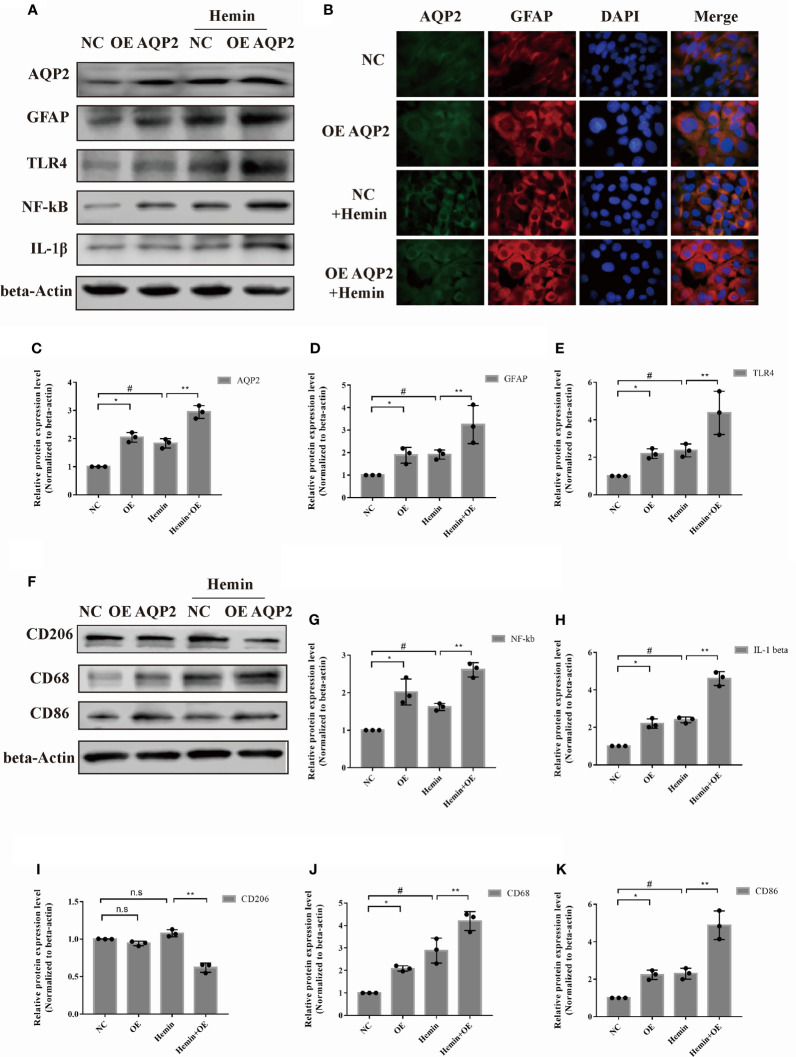
AQP2 overexpression directly increases astrocyte activation and indirectly promotes microglia migration. **(A)** Astrocyte were transfected with pcDNA3.1 empty vector or pcDNA3.1-AQP2 plasmid, and AQP2, GFAP, TLR4, NF-kB and IL-1β were assessed by western blotting (n = 3/group). **(B)** Immunofluorescence of AQP2 (red signal) and GFAP (green signal) in astrocyte with AQP2 overexpression or negative control (empty vector). Astrocyte cell nuclei were immunostained (blue, DAPI). Scale bar: 10 μm. **(C, D)** and **(G, H)** Quantification of the relative protein expression levels of AQP2, GFAP, TLR4, NF-kB and IL-1β. (^*^p < 0.05 vs. empty vector group; ^**^p < 0.05 vs. empty vector group exposed to hemin; ^#^p < 0.05 empty vector group vs. empty vector group exposed to hemin). The data are presented as the mean ± s.d. of three independent experiments. **(F)** Microglia were treated with medium of astrocyte for 24h transfected with pcDNA3.1 empty vector or pcDNA3.1-AQP2 plasmid (with or without hemin). CD206, CD68 and CD86 were assessed by western blotting (n = 3/group). Quantification of the relative protein expression level of CD206 **(I)**, CD68 **(J)** and CD86 **(K)** is shown. (^*^p < 0.05 vs. empty vector group; ^**^p < 0.05 vs. empty vector group exposed to hemin; ^#^p < 0.05 empty vector group vs. empty vector group exposed to hemin). The data are presented as the mean ± s.d. of three independent experiments.

Based on the cell viability result, 50umol/L hemin was selected for further investigation (**Supplementary Table 1**). To investigate the potential role of AQP2 in astrocyte activation, we treated astrocytes with an AQP2-overexpression plasmid and cultured them with or without hemin treatment (**Figures 4A, C**). We observed a moderate increase in TLR4, NFκB (p65), GFAP, and IL-1β expression in AQP2 overexpression-stimulated astrocytes compared to the NC group (p < 0.05, vs. empty vector transfection groups). (**Figures 4D, E** **, G, H**). The same result was also found in the AQP2 overexpression group treated with hemin compared to the NC group treated with hemin (**Figure 4B**). We then tested whether the supernatant from AQP2-upregulated astrocytes could activate the microglia. We treated rat microglia with conditioned media from AQP2 overexpression-stimulated astrocytes. Interestingly, conditioned media from AQP2-upregulated astrocytes increased CD86 and CD68 levels in the microglia (**Figures 4J, K**, p < 0.05, vs. empty vector transfection groups), along with decreased CD206 levels in the AQP2 overexpression group treated with hemin compared to the NC group treated with hemin (p < 0.05) (**Figures 4** **F, I**). In addition, we observed an increased M1/M2 phenotype in rat microglia treated with conditioned media from AQP2 overexpression-stimulated astrocytes treated with hemin compared to the NC group treated with hemin (p < 0.05) (**Supplementary Figure 1D, E**). Morphological observation showed that the morphology of microglia cells changed from round shape to multiple ramified shape and enlarged appearance after treatment with the supernatant from AQP2-upregulated astrocytes in microglia cells (**Supplementary Figure 1F**).

**Figure 5:**
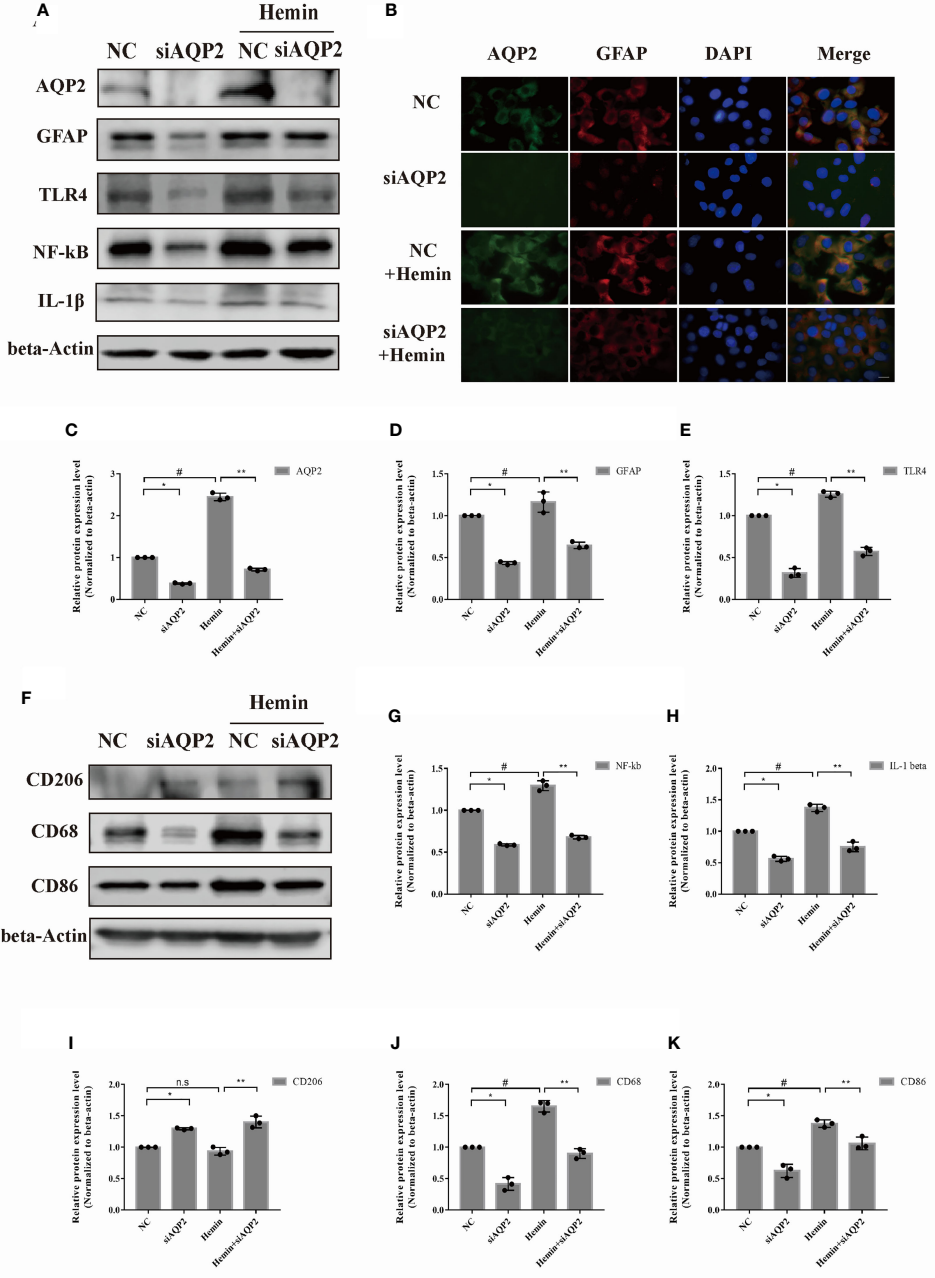
AQP2 silencing directly inhibit astrocyte activation and indirectly reduce microglia migration. **(A)** Astrocyte were transfected with siAQP2 or siRNA-NC, and expression of AQP2, GFAP, TLR4, NF-kB and IL-1β were assessed by western blotting (n = 3/group). **(B)** Immunofluorescence of AQP2 (red signal) and GFAP (green signal) in astrocyte in astrocyte treated with siAQP2 or siRNA-NC. Astrocyte cell nuclei were immunostained (blue, DAPI). Scale bar: 10 μm. **(C–E, G, H)** Quantification of the relative protein expression levels of AQP2, GFAP, TLR4, NF-kB and IL-1β. (^*^p < 0.05 vs. siRNA-NC group; ^**^p < 0.05 vs. siRNA-NC exposed to hemin; ^#^p < 0.05 siRNA-NC group vs. siRNA-NC group exposed to hemin). The data are presented as the mean ± s.d. of three independent experiments. **(F)** Microglia were treated with medium of astrocyte for 24h transfected with siRNA or si-AQP2 RNA (with or without hemin). CD206, CD68 and CD86 were assessed by western blotting (n = 3/group). Quantification of the relative protein expression level of CD206 **(I)**, CD68 **(J)** and CD86 **(K)** is shown. (^*^p < 0.05 vs. siRNA-NC group; ^**^p < 0.05 vs. siRNA-NC exposed to hemin; ^#^p < 0.05 siRNA-NC group vs. siRNA-NC group exposed to hemin). The data are presented as the mean ± s.d. of three independent experiments.

We also examined the effect of AQP2 silencing on astrocyte activation using siAQP2. Based on the silencing efficiency of the three types of siRNAs, we chose si-AQP2-3 for further study (**Figures 5A, C**, **Supplementary Figure 1G**). We observed reduced levels of TLR4, NF-κB (p65), GFAP, and IL-1β expression in AQP2 silence-stimulated astrocytes compared to the NC group (p < 0.05, vs. siRNA-NC transfection groups). (**Figures 5D, E** **, G, H**). The administration of hemin to siAQP2- or siRNA-NC-transfected astrocytes showed similar results (**Figure 5B**, p < 0.05). Conditioned media from AQP2 silenced astrocytes decreased CD86 and CD68 in microglia (**Figures 5J, K** **;** p < 0.05, vs. siRNA-NC transfection groups), along with increased CD206 in the AQP2-silenced group treated with hemin compared with that in the NC group treated with hemin (p < 0.05) (**Figures 5** **F, I**). In addition, the M1/M2 phenotype was decreased in rat microglia treated with conditioned media from AQP2 silence-stimulated astrocytes treated with hemin compared to the NC group treated with hemin (p < 0.05) (**Supplementary Figure 1H, I**). Together, these results demonstrated that AQP2 participates in the inflammatory response in a hemin-treated ICH model *in vitro*.

### AQP2 Mediates Hemin-Induced Inflammatory Response by Targeting the TLR4/NFκB Signaling Pathway

**Figure 6:**
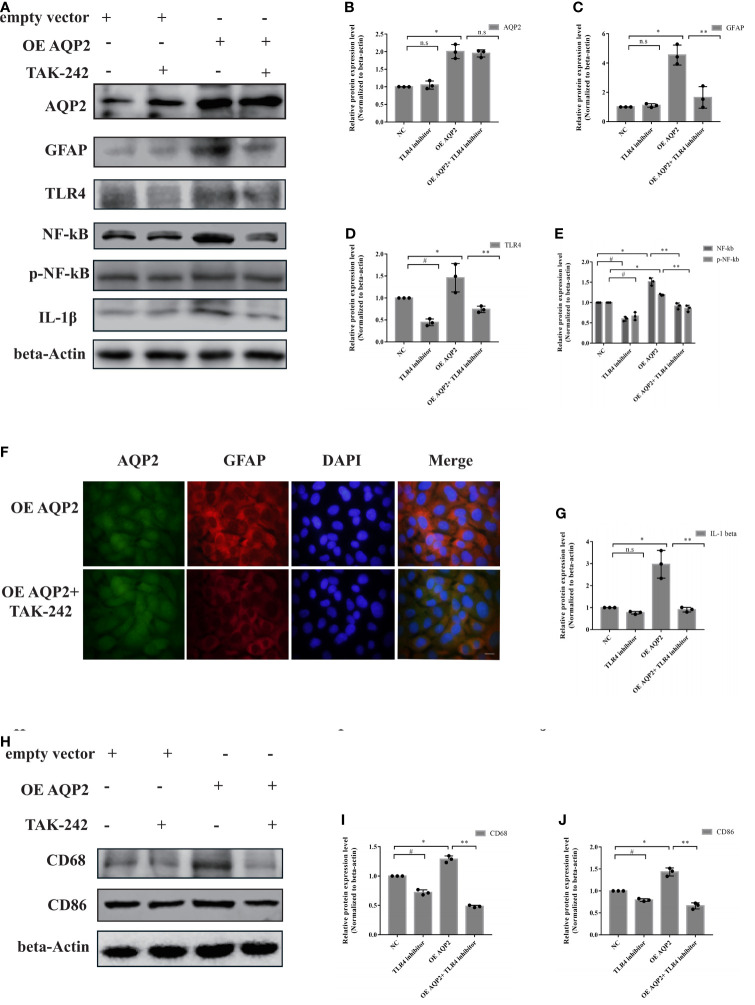
AQP2 overexpression-induced pro-inflammation secretion is partially caused by TLR4 signaling activation in astrocyte. Astrocyte with overexpressed AQP2 (pcDNA3.1-AQP2 plasmid) were exposed to TLR inhibitor (TAK-242) for 24 h. **(A)** Western blot analysis of AQP2, GFAP, TLR4, NF-κB, phospho- NF-κB and IL-1β in astrocyte transfected with pcDNA3.1 empty vector or pcDNA3.1-AQP2 plasmid treated with or without TAK-242 (n = 3/group). **(B–E, G)** Quantification of the relative protein expression levels of AQP2, GFAP, TLR4, NF-kB and IL-1β. (^#^p < 0.05, empty vector + vehicle vs. empty vector+ TAK-242; ^*^p < 0.05, empty vector + vehicle vs. pcDNA3.1-AQP2 plasmid + vehicle; ^**^p < 0.05, pcDNA3.1-AQP2 plasmid + vehicle vs. pcDNA3.1-AQP2 plasmid + TAK-242). The data are presented as the mean ± s.d. of three independent experiments. **(F)** Immunofluorescence of AQP2 (red signal) and GFAP (green signal) in astrocyte with AQP2 overexpression treated with or without TAK-242. Astrocyte cell nuclei were immunostained (blue, DAPI). Scale bar: 10 μm. **(H)** Microglia were treated with medium of astrocyte for 24h transfected with pcDNA3.1 empty vector or pcDNA3.1-AQP2 plasmid (with or without TAK-242). CD68 and CD86 were assessed by western blotting (n = 3/group). Quantification of the relative protein expression level of CD68 **(I)** and CD86 **(J)** is shown. (^#^p < 0.05, empty vector + vehicle vs. empty vector+ TAK-242; ^*^ p < 0.05, empty vector + vehicle vs. pcDNA3.1-AQP2 plasmid + vehicle; ^**^p < 0.05, pcDNA3.1-AQP2 plasmid + vehicle vs. pcDNA3.1-AQP2 plasmid + TAK-242). The data are presented as the mean ± s.d. of three independent experiments.

Given the key role of AQP2 in the regulation of inflammatory responses in astrocytes, we investigated the potential role of the TLR4/NFκB-p65 signaling pathway in AQP2-induced inflammation. AQP2 was successfully overexpressed in astrocytes after transfection with an AQP2 overexpression plasmid (**Figures 6A, B**), which was later used to assess the effects of TLR4 inhibitor administration. TLR4/phospho-NFκB-p65 was inhibited in the TLR4 inhibitor + NC plasmid transfection and TLR4 inhibitor + AQP2 plasmid transfection groups (**Figures 6** **D, E**). The treatment of astrocytes with a TLR4 inhibitor did not affect astrocyte activation or IL-1β secretion. AQP2 plasmid transfection-induced astrocyte activation and IL-1β secretion were reduced by the TLR4 inhibitor in the TLR4 inhibitor + AQP2 plasmid transfection group (**Figures 6** **B, C, F, G**) (p < 0.05, vs. vehicle + AQP2 plasmid transfection groups). CD86 and CD68 expression levels were lower in the TLR4 inhibitor + NC plasmid group than in the vehicle + NC plasmid group (P < 0.05). Similar alterations in CD68 and CD86 were also observed in the TLR4 inhibitor + AQP2 plasmid group compared to the vehicle + AQP2 plasmid group (**Figures 6** **H–J**) (p < 0.05). Thus, activation of the TLR4/NFκB-p65 signaling pathway mediated AQP2 to promote a increase in the inflammatory response in astrocytes.

## Discussion

Blood components derived from hematomas induce inflammation following ICH. Microglial and astrocyte activation and related inflammatory mediator secretion are key contributors to the pathophysiological mechanisms of secondary brain injury and brain edema after ICH (21). AQPs, as astrocyte water channels, play a role in edema formation, astrocyte migration, and modulation of inflammation (22, 23). AQP4 is the most studied AQP in the CNS and is mainly distributed in astrocytes. AQP4 contributes to the development of inflammatory demyelination diseases of the CNS (neuromyelitis optica spectrum disorder [NMOSD] or acute disseminated encephalomyelitis [ADEM]) (24) and promotes brain inflammation in neurodegenerative diseases (25), stroke (26), and other disorders (27). AQP4 is also involved in astrocyte-microglia communication when water passes through the lamellipodium and into the cytoplasm *via* an osmotic gradient (28, 29). Similar to AQP4, AQP2 not only influences fluid transport in many epithelial and endothelial tissues (30), but also participates in the regulation of inflammation and apoptosis in renal diseases. AQP2 is distributed in the CNS [e.g., the ependymal cell layer, subcortical white matter, and hippocampus (31)] and peripheral nervous system [e.g., rat extra-temporal facial nerve (20), rat trigeminal ganglion neurons (32), and small-diameter dorsal root ganglia neurons (33)]. In the peripheral nervous system, AQP2 may be involved in pain transmission and neuropathic nerve injury (34). However, AQP2 function and regulation and its localization in specific CNS cells remain unknown.

The novelty of our study was that AQP2 levels in the serum were lower in patients with ICH than in healthy controls. Further analysis revealed that lower AQP2 levels were related to sex and elevated blood neutrophil counts on the first day post-ICH. The blood neutrophil count is correlated with systemic inflammation. Additionally, patients with favorable outcomes (mRS score ≤3) had higher serum AQP2 concentrations than those with poor outcomes (mRS score>3). Therefore, AQP2 may be related to prognosis and inflammation injury after ICH, and may be a potential biological serum marker of inflammation in early-stage ICH.

AQP2 was partly localized in astrocytes labeled with GFAP and upregulated simultaneously with GFAP in the collagenase-induced hemorrhage rat brain. In this study, AQP2 overexpression in astrocytes in the brain or peripheral blood cells corresponded to a reduction in AQP2 excretion or degradation in the cerebrospinal fluid or serum. It is probably easier to make an analogy in a hypertensive rat study by Zhao et al., in which imidapril reduced urinary reabsorption and showed an antihypertensive effect *via* the downregulation of AQP2 expression in renal tissues, accompanied by increased AQP2 excretion in the urine (35). The expression of AQP2 in cerebrospinal fluid or blood cells of ICH patients requires further study, as well as observation of AQP2 function by culturing lymphocytes derived from ICH patients *in vitro*.

Few studies have assessed the role of AQP2 in the CNS. However, the mechanisms involved in AQP2 modulation require further investigation. As mentioned above, the main components of secondary injury after ICH include inflammation, oxidative stress, excitotoxicity, and cytotoxicity (4). A previous study showed that AQP2 expression is sensitive to cellular reactive oxygen species (ROS) and intracellular calcium levels in renal cells (36). NADPH oxidase (NOX)-derived ROS results in oxidative stress and enhances AQP2 transcription by decreasing cAMP hydrolysis in renal collecting duct principal cells (PCs) (37). NLRP3 deficiency prevents AQP2 downregulation in renal epithelial cells and is involved in inflammation regulation in mice fed a high-fat diet (14). Therefore, the ICH-induced overload of cellular ROS and an excessive inflammatory response may contribute to AQP2 overexpression in astrocytes and enhance AQP2 transcription.

Given the decreased serum AQP2 levels in ICH patients, which correlated with blood neutrophil count and prognosis, the co-localization and upregulation of AQP2 and GFAP in ICH-induced SD rats suggested the important role of AQP2 in ICH-induced astrocyte activation. Moreover, astrocytes and microglia are critical immune cells in neuroinflammation after ICH, highlighting the potential role of AQP2 in astrocytes and inflammatory responses. Our further investigation showed that AQP2 upregulation in astrocytes promotes the expression of TLR4 and NFκB-p65, and the increase in NFκB-p65 was more significant than that of TLR4. Glial cells have low basal NF-κB activity, but are highly inducible (38) and play a decisive role in causing inflammation in the brain (39, 40). The performance trends of TLR-4 and NF-κB are not consistent, which may be derived from various triggers of the canonical NF-κB pathway. In addition to cell surface receptors such as toll-like receptors (TLRs), proinflammatory cytokines and T cell receptors or B cell receptors can trigger NF-κB pathway as well (41, 42). In addition, GAFP levels and pro-inflammatory cytokine IL-1β secretion increased after AQP2 overexpression. This result is consistent with those of previous studies reporting that AQP2 influences not only fluid transport but also cytokine release (e.g., IL-6, TNFα, IL-1β) (13). TLR4, an essential member of the TLR family involved in innate immunity, is mainly expressed in astrocytes or microglia and contributes greatly to cytokine production after ICH (43). Our findings demonstrate that a TLR4-inhibitor blocked the positive regulatory effect of AQP2 on GFAP and IL-1β in astrocytes. These results suggest that AQP2 participates in ICH-induced inflammatory injury through astrocyte activation and cytokine release, mediated by the TLR4/NFκB-p65 signaling pathway. Moreover, silencing of AQP2 inhibited the TLR4/NFκB-p65 signaling pathway and reduced IL-1β secretion in hemin-treated astrocytes. A previous study demonstrated that inhibition of the TLR4/NFκB-p65 signaling pathway alleviated neurological function and cerebral edema and repressed neuronal apoptosis in ICH rats (44). Therefore, AQP2 may be a therapeutic target for reducing ICH-induced inflammatory response.

AQP2 also promotes microglial polarization *via* astrocyte-microglia crosstalk. Microglia and macrophages can be divided into two phenotypes. The M1 phenotype, marked with CD86, CD68, etc., is considered a pro-inflammatory/injury-inducer and produces inflammatory cytokines (IL-1β, IL-12, IL-23, and TNF-α), whereas the M2 phenotype, marked with CD206, CD36, etc., is anti-inflammatory and promotes the overall regeneration of tissue and resolution of inflammation (3). We found that CD68 and CD86 were upregulated with higher hemin concentrations (25-75µmol/L), while CD206 was also increased in primary microglia treated with 50 and 75 µmol/L hemin or in the HAPI cell line treated with 75µmol/L. This may be due to the suppression of excessive immune response and inflammation injury. However, the ratio of the M1/M2 phenotype was increased, reflecting a hemin-induced pro-inflammatory response in general. A similar observation was also reported by Shtaya et al., showing that an anti-inflammatory (repair) process occurs in parallel with neuroinflammation in patients with clinical ICH (45). Furthermore, we observed polarization into the pro-inflammatory M1 phenotype, reflected by upregulation of CD86 and CD68 in HAPI cultured in the medium of astrocytes treated with an AQP2-overexpression plasmid. This may be because AQP2-induced astrocyte overactivation produces proinflammatory cytokines in the culture medium, which may evoke the M1/M2 phenotype switching in microglia after ICH. Astrocyte-secreted proinflammatory cytokines can lead to changes in microglia/macrophage subtypes. Astrocyte-derived IL-15 skewed microglia toward a proinflammatory phenotype after ICH (10). In addition to IL-15, astrocyte-derived IL-33 (a member of the IL-1 family) strengthens microglial function (9) and promotes microglial transformation from the M1 to M2 phenotype in brain tissues after ICH (46). Therefore, AQP2 may indirectly induce microglial polarization to the M1 phenotype, which may further augment the pro-inflammatory response after ICH.

In summary, the results of our study demonstrate that AQP2 may be a biological serum marker of inflammation in the early stages of ICH. AQP2 overexpression induces astrocyte activation and pro-inflammatory secretion *via* the TLR4/NFκB-p65 signaling pathway. In addition, AQP2 participates in astrocyte-microglia crosstalk and indirectly induces microglial polarization into the M1 phenotype in HAPI, which may augment the pro-inflammatory response after ICH. Based on the pro-inflammatory role of AQP2 in glial cells, we assumed that lower expression of AQP2 in peripheral serum might indirectly indicate lower AQP2 excretion but higher AQP2 expression in peripheral blood cells or CNS, which aggravates central immune microenvironment dysfunction after a hemorrhagic stroke along with poor prognosis. Therefore, therapies targeting AQP2 may alleviate neurological injury following ICH. Although our data implicated AQP2 as a driver of the pro-inflammatory response of astrocytes following ICH, the extent to which AQP2 affects inflammatory injury warrants further investigation.

### Limitations

In this study, serum AQP2 levels were correlated with inflammation parameter blood neutrophil count and 90-day mRS scores, but not with ICH severity (NIHSS score) on admission, which may have resulted from the small sample size. Larger prospective studies are required to verify this finding. Data from weekly head CT re-examination and additional blood tests for AQP2 and clinical parameters should be obtained on days 4, 7, 10, and 14 after ICH onset.

### Prospects

The potential role of AQP2 in pro-inflammatory response sheds light on the future selection of antihypertensive drugs. Imidapril can reduce urinary reabsorption and provide an anti-hypertensive effect by downregulating AQP2 expression in renal tissues, which may indirectly provide mediation by reducing the stimulatory effect of angiotensin II (AngII) on the expression of vasopressin-V2 receptor mRNA (35). Therefore, ACE inhibitors, among the best-tolerated antihypertensive drugs, not only have a hypotensive effect, but also negatively regulate AQP2 expression in astrocytes after ICH. However, the assessment of the benefits of treatment with ACE inhibitors on the prognosis of ICH requires further clinical trials.

## Data Availability Statement

The original contributions presented in the study are included in the article/**Supplementary Materials**. Further inquiries can be directed to the corresponding authors.

## Ethics Statement

The studies involving human participants were reviewed and approved by the Ethics Committee of the Second Xiangya Hospital of Central South University (approval number: LYZ2020008). The patients/participants provided their written informed consent to participate in this study. The animal study was reviewed and approved by the Central South University Animal Experimentation Committee (approval number: 2021013). Written informed consent was obtained from the individual(s) for the publication of any potentially identifiable images or data included in this article.

## Author Contributions

SD drafted the manuscript. QL and XC conducted a literature review. WL contributed to and finalized the draft. All authors have read and approved the final manuscript.

## Funding

This study was funded by the Natural Science Foundation of Hunan Province (#2020JJ4798).

## Conflict of Interest

The authors declare that the research was conducted in the absence of any commercial or financial relationships that could be construed as potential conflicts of interest.

## Publisher’s Note

All claims expressed in this article are solely those of the authors and do not necessarily represent those of their affiliated organizations, or those of the publisher, the editors and the reviewers. Any product that may be evaluated in this article, or claim that may be made by its manufacturer, is not guaranteed or endorsed by the publisher.
