# Supplementary material for: AQP2 Promotes Astrocyte Activation by Modulating the TLR4/NFκB-p65 Pathway Following Intracerebral Hemorrhage
Source: Front Immunol. 2022 Mar 21;13:847360. doi: 10.3389/fimmu.2022.847360 (PMC8978957; doi:10.3389/fimmu.2022.847360)
Supplement: Supplementary file 1 [file DataSheet_1.docx]

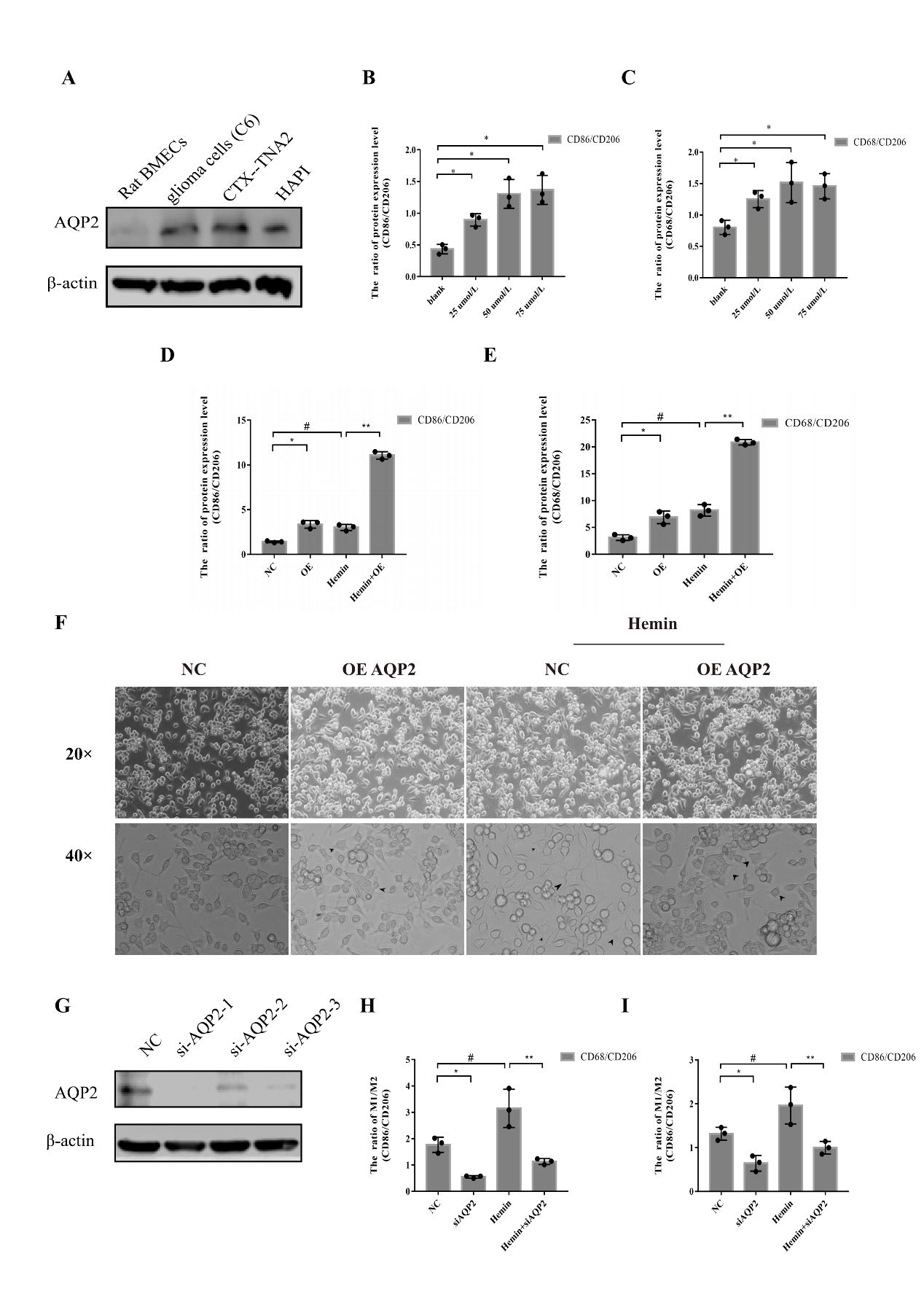


**Supplementary Figure 1. M1/M2 phenotype of microglia cultured with the medium of astrocyte.** **A.** Western blotting of AQP2 in different types of cell lines. **B and C.** Quantification of the M1/M2 phenotype of microglia cultured with medium of astrocyte treated with different concentrations of hemin (25, 50 and 75 umol/l) (^*^p < 0.05, vs blank group). **D and E.** Quantification of the M1/M2 phenotype of microglia cultured with medium of AQP2 overexpression-stimulated astrocyte. (^*^p < 0.05, empty vector vs pcDNA3.1-AQP2 plasmid; ^#^p < 0.05, empty vector + vehicle vs empty vector + hemin; ^**^ p < 0.05, empty vector + hemin vs pcDNA3.1-AQP2 plasmid + hemin). The data are presented as the mean ± s.d. of three independent experiments. **F.** Morphological observation in microglia cells treated with the supernatant from AQP2-upregulated astrocytes. **G.** The efficacy of siAQP2 on silencing the expression of AQP2 in astrocyte. **H and I.** Quantification of the M1/M2 phenotype of microglia cultured with medium of AQP2 silence-stimulated astrocyte. (^*^ p < 0.05, siRNA-NC vs si-AQP2; ^#^p < 0.05, siRNA-NC + vehicle vs siRNA-NC + hemin; ^**^ p < 0.05, siRNA-NC + hemin vs si-AQP2 + hemin)
