# Supplementary material for: AQP2 Promotes Astrocyte Activation by Modulating the TLR4/NFκB-p65 Pathway Following Intracerebral Hemorrhage
Source: Front Immunol. 2022 Mar 21;13:847360. doi: 10.3389/fimmu.2022.847360 (PMC8978957; doi:10.3389/fimmu.2022.847360)
Supplement: Supplementary file 2 [file DataSheet_2.docx]

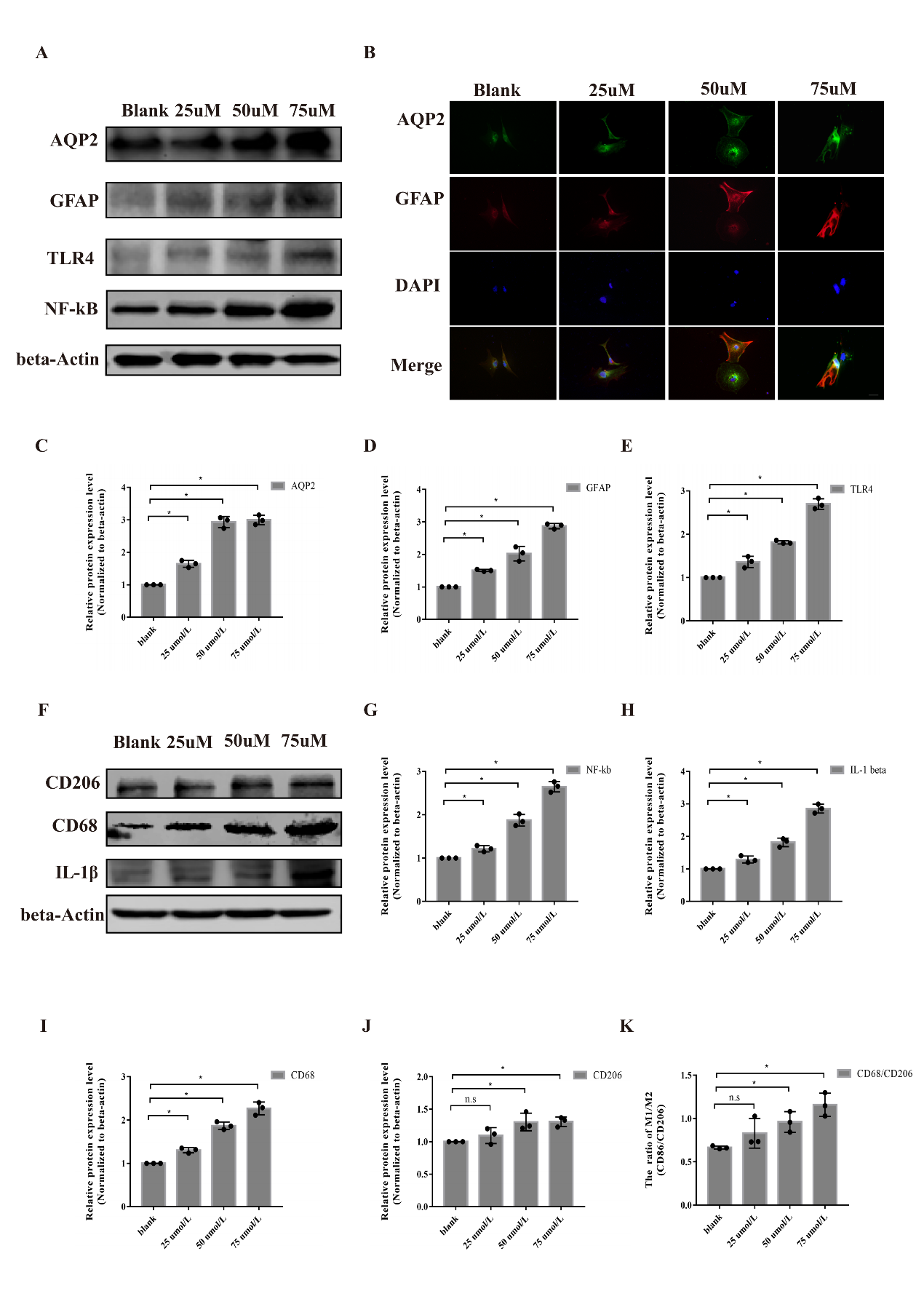


**Supplementary Figure 2. Hemin induced primary astrocyte activation and microglia polarization**. Primary astrocyte was exposed to hemin for 12h. A. AQP2, GFAP, TLR4, NF-kB and IL-1β protein levels in astrocyte were assessed by western blotting in the blank control group, and different groups were treated with several concentrations of hemin (n = 3/group). B. Primary astrocyte exposed to hemin for 12h were stained with antibodies to AQP2 (green signal) and visualized by immunofluorescence microscopy. Astrocyte were immunostained for nuclei (blue, DAPI). Representative images are shown. Scale bar: 10 μm. Quantification of the relative protein expression level of AQP2(C), GFAP (D), TLR4 (E), NF-kB (G) and IL-β (H) after 12 h hemin treatment. *p < 0.05 vs. the blank group without hemin. F. CD206 and CD68 protein levels in microglia in the blank control group were assessed by western blotting, and different groups were treated with several concentrations of hemin (n = 3/group). Quantification of the relative protein expression level of CD206 (I) and CD68 (J) are shown. K. Quantification of the M1/M2 phenotype of primary microglia cultured with medium of astrocyte treated with different concentrations of hemin (25, 50 and 75 umol/l). ^*^p < 0.05 vs. the blank group without hemin. The data are presented as the mean ± s.d. of three independent experiments.
