# Supplementary figures and images for: AQP2 Promotes Astrocyte Activation by Modulating the TLR4/NFκB-p65 Pathway Following Intracerebral Hemorrhage
Source: Front Immunol. 2022 Mar 21;13:847360. doi: 10.3389/fimmu.2022.847360 (PMC8978957; doi:10.3389/fimmu.2022.847360)

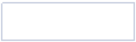

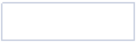

Supplement: Supplementary file 3 [file Table_1.pdf]
